# Clinical value of miR-135 and miR-20a combined with multi-detector computed tomography in the diagnosis of gastric cancer

**DOI:** 10.1186/s12957-021-02395-z

**Published:** 2021-09-18

**Authors:** Wenwen Han, Xiangzhen Bu, Yanli Liu, Fang Liu, Yujie Ren, Yongsheng Cui, Shuhong Kong

**Affiliations:** 1 Department of CT Room, Dongying People’s Hospital, No. 317 NanYi Road, Dongying, 257091 China; 2 Department of Radiology, Dongying District People’s Hospital, Dongying, 257000 China; 3 Health Care Department, Dongying People’s Hospital, Dongying, 257091 China; 4 Department of Oncology, Dongying People’s Hospital, Dongying, 257091 China; 5 Department of CT Examination, Shengli Oilfield Central Hospital, Dongying, 257000 China

**Keywords:** Gastric cancer, miR-135, miR-20a, Multi-detector computed tomography, Combined diagnosis

## Abstract

**Background:**

To study the clinical value of miR-135 and miR-20a combined with multi-detector computed tomography (MDCT) in the diagnosis of gastric cancer (GC).

**Method:**

A total of 146 patients with GC admitted to our hospital from January 2017 to June 2019 were selected and enrolled in the GC group. Another 103 patients with gastritis received in the same period were selected for the non-GC group. Besides, 95 healthy subjects who received physical examination in our hospital were selected into the healthy control group. Real-time fluorescence quantitative polymerase chain reaction (qRT-PCR) was used to detect the expression of serum miR-135 and miR-20a for each group. MDCT was used for detecting the clinical staging map of the enrolled patients. Pearson’s correlation analysis was used to analyze the correlation between serum miR-135 and miR-20a in patients with GC. The receiver operating characteristic (ROC) curve was drawn to analyze value of miR-135 and miR-20a in the diagnosis of GC.

**Results:**

Compared with non-GC group and healthy control group, the levels of serum miR-135 and miR-20a increased significantly in the GC group, while no significant difference was found between non-GC group and healthy control group (*P* > 0.05). Analysis of the relationship with clinical characteristics showed that the expression of serum miR-135 and miR-20a in the GC group was significantly correlated with the progression of GC, TNM stage, degrees of differentiation, status of lymph node metastasis, and distant metastasis (*P* < 0.01). Pearson’s correlation analysis results showed positive correlations between miR-135 and miR-20a (*r* = 0.634, *P* = 0.000). The ROC analysis results showed that the optimal diagnostic values of miR-135 and miR-20a for GC were 7.56 and 5.82 respectively. The area under the curve (AUC) was 0.873 and 0.793 respectively. The 95% confidence interval (CI) was 0.811-0.935 and 0.697-0.890 respectively. The sensitivity and specificity of miR-135 and miR-20a combined with MDCT in the diagnosis of GC were 90.41% and 93.20% respectively. The sensitivity of combined use was significantly higher than that of single detection (*P* < 0.01).

**Conclusion:**

There are high expression levels of serum miR-135 and miR-20a in patients with GC. A combined detection of miR-135 and miR-20a with MDCT can improve the diagnostic sensitivity of GC and improve the accuracy of the final diagnosis. Therefore, multiple combined detection is valuable in the diagnosis of GC.

## Background

Gastric cancer or gastric carcinoma (GC) is a common malignant tumor of the digestive system, which originates from the mucosal epithelial cells of the gastric wall at first. GC has a higher incidence in the middle-aged and elderly people, and also significantly higher rate in males than that in females [1, 2]. According to relevant studies [3], the morbidity and mortality of GC rank the fifth and third globally in malignant tumors respectively. In China, its morbidity and mortality ranks the second and third of all malignant tumors, much higher than the world average. Patients at the early stage of GC may experience a better prognosis and higher cure rate. However, patients in the early stage usually have none obvious clinical symptoms, only a few patients show indigestion, fullness, discomfort, and other common symptoms [4, 5]. As a result, there is a high risk of missed diagnosis and hence patients may loss the best treatment opportunity. Consequently and unluckily, patients may have entered the clinical middle-late stage at the time of reexamination, resulting in poor prognosis, high probability of distal metastasis and recurrence, and reduced survival time accordingly [6]. Therefore, improvement in the detection rate of early GC is of great importance for the treatment and prognosis of GC patients. At present, biopsy, gastroscopy, and some tumor markers have been used for diagnosing patients with GC [7–9]. However, biopsy is invasive, and the existing tumor markers exhibit unsatisfied sensitivity and specificity, highlighting the necessity to find other safe and efficient diagnostic methods [10].

Micro ribonucleic acid (microRNA or miRNA) is a non-coding micromolecule RNA, widely existing in cells, which is involved in the regulation of cell proliferation, differentiation, apoptosis, and metabolism [11, 12]. Multiple miRNAs are abnormally expressed in malignant tumors that may promote or inhibit the occurrence of tumors [13, 14]. Studies by Chao C et al. have shown that miR-135 plays an important role in the occurrence and development of digestive system cancer, breast cancer, and prostate cancer [15–17], but whether its expression in gastric cancer is abnormal is still rarely reported. The diagnostic value of gastric cancer cannot be determined for the time being. The research of Yang R et al. showed that miR-20a is a promising biomarker for gastric cancer, and its expression in the serum of gastric cancer patients and healthy people is significantly different. This study wants to further demonstrate from the facts [18, 19]. Meanwhile, general CT scan has some limitations in detecting preoperative staging of GC, while multi-phase can by using MDCT that exhibits better effect in evaluating tumor size, depth of invasion, and extent of lymph node metastasis, which has been concerned about gradually by clinicians [20]. However, it still has disadvantage in the display of gastric wall structures [21]. In view of the above interpretation, the present study was carried out with the inclusion of 146 patients with GC admitted to the hospital from January 2017 to June 2019, with the purpose to investigate the clinical value of serum miR-135 and miR-20a combined with MDCT in the diagnosis of GC.

## Data and methods

### General data

A total of 146 patients with GC admitted to our hospital from January 2017 to June 2019 were included in the GC group, including 97 males and 49 females, aged 32-68 years, with an average age of 53.04 ±11.37 years. Inclusion criteria: Patients with GC diagnosed by operation or pathological examination; patients who did not receive any treatment before detection; patients who could cooperate with this study and whose medical records were complete. Exclusion criteria: Patients with malignant tumors or blood diseases; and patients with a long history of drug dependence. A total of 103 patients without GC in the same period were included in the non-GC group, including 62 males and 41 females, aged 29-70 years, with an average age of 49.56 ± 12.81 years. In addition, 95 healthy subjects received physical examination in our hospital were selected into the healthy control group, including 53 males and 42 females, aged 27-66 years, with an average age of 46.28 ± 9.54 years. No statistically significant difference was found in general data of the three groups of patients. This study was approved by the Ethics Committee of the hospital. All patients and their families were informed of the study and provided written informed consent forms.

### Main reagents and instruments

Serum microRNA rapid extraction kit (GENMED Pharmaceutical Technology Co., Ltd., Shanghai); real-time fluorescence quantitative polymerase chain reaction (qRT-PCR) kit (Takara); Moloney murine leukemia virus reverse transcriptase (Takara); ELISA kit (R&D Systems, USA); synthesis of serum primers (TIANGEN Biotech Co., Ltd., Beijing); ultraviolet spectrophotometer (Shanghai Lab-Spectrum Instruments Co., Ltd.); PCR instrument (ABI, USA). Raceanisodamine hydrochloride injection [national medicine permission number (NMPN) H41023400; Sinopharm Group Rongsheng Pharmaceutical Co., Ltd.]; 64-detector dual-source multi-slice CT machine (SIEMENS, Germany); iopromide injection (NMPN, H10970166; Schering Pharmaceutical Limited).

### Test methods

#### Preparation of samples

On the 2nd morning, elbow venous blood was taken from the enrolled subjects on an empty stomach. Collected samples were centrifuged at 3500 rpm for 10 min at room temperature. The obtained supernatant was then divided into two equal parts and stored in dry test tubes at −80 T-PCR) further use.

#### Detection of serum miR-135 and miR-20a expressions by qRT-PCR

One of the samples was used for separating total RNA by RAN extraction reagent, and the RNA purity was detected by ultraviolet spectrophotometer. The expression levels of miR-135 and miR-20a were detected by qRT-PCR. The primers are shown in Table 1. The amplification conditions were as follows: Pre-degeneration at 95 and the RNA and extension at 72 with 45 cycles of amplification. The experiment was repeated for 3 times. The relative expression levels of serum indexes (ΔCt = Ct target gene −CtU6) were calculated by 2^−ΔΔ Ct^.

**Table 1:** List of primers for detection of serum miR-135 and miR-20a

Factors	Upstream primer	Downstream primer
miR-135	5′-ATGTACGCTACTGTGAGCTG-3′	5′-GTCAGCGAGTGAGCATAG-3′
miR-20a	5′- GCGGCGGTAAAGTGCTTATAGTG-3′	5′-TGCAGGGTCCGAGGTAT-3′
U6	5′-CCCTCCAGAGAGCGTTAT- GTGA-3′	5′-GTTTCTGAAAATTA-CAGGGTCATTTGTG-3′

#### MDCT examination

Patients were informed to be fasted for 8 h before the examination, and had 600-1000 mL drinking water 20 min before the examination, and received an intramuscular injection of 20 mg anisodamine. Patients were adjusted to keep their supine, prone or lateral position, and CT scan was performed first, followed by intravenous injection of iopromide contrast agent through the elbow vein (1.5 mL/kg at 3.0 mL/s). Scans were performed in 30 s (arterial phase), 60~70 s (venous phase), and 3~4 min (equilibrium phase) after the injection of contrast agent. Then, images were acquired and observed for damage of the adjacent tissue of lesion, as well as liver and distal metastasis. The scan ranged from the umbilical plane to the top of the mediastinum. Scanning parameters included voltage at 120 kV, current of 250-300 mA, slice thickness of 5 mm, and pitch of 1.25 mm.

### Criterion for determination of results

Staging of enrolled patients was performed according to the TNM staging standard of patients with GC in the American Joint Commission on Cancer (AJCC) 7th version [22]. Among them, *T* refers to the depth of invasion (*T*0, no tumor in the excised specimen; *T*1, invasion in the laminae propria mucosae, muscularis mucosa, or submucosa; *T*2, invasion in lamina propria; *T*3, invasion in subserosa connective tissue, non-invasion in the adjacent structure; and *T*4, invasion both in subserosa and the adjacent structure). When the expression of serum miR-135 and miR-20a exceeds the critical value, it is positive; if equal to or lower than the critical value, it is negative. In case that one or more items of the combined detection method is positive, the result is positive; if all of the items are negative, the result is negative.

### Statistical method

The SPSS20.0 was used for data processing and analysis. The measurement data with normal distribution were expressed by χ ± *s*, and the independent sample *t* test was used for the comparison between groups. Pearson’s correlation analysis was used to analyze the correlation between serum miR-135 and miR-20a in patients with GC. The receiver operating characteristic (ROC) curve was used for analysis of the value of miR-135 and miR-20a in diagnosis of GC. Sensitivity = true positive number/(true positive number + false negative number) × 100%; specificity = true negative number/(true negative number + false positive number) × 100%. *P* < 0.05 means the difference is statistically significant.

## Results

### Comparison of serum miR-135 and miR-20a expression levels in each group and their relationships with clinical characteristics of GC patients

**Fig. 1:**
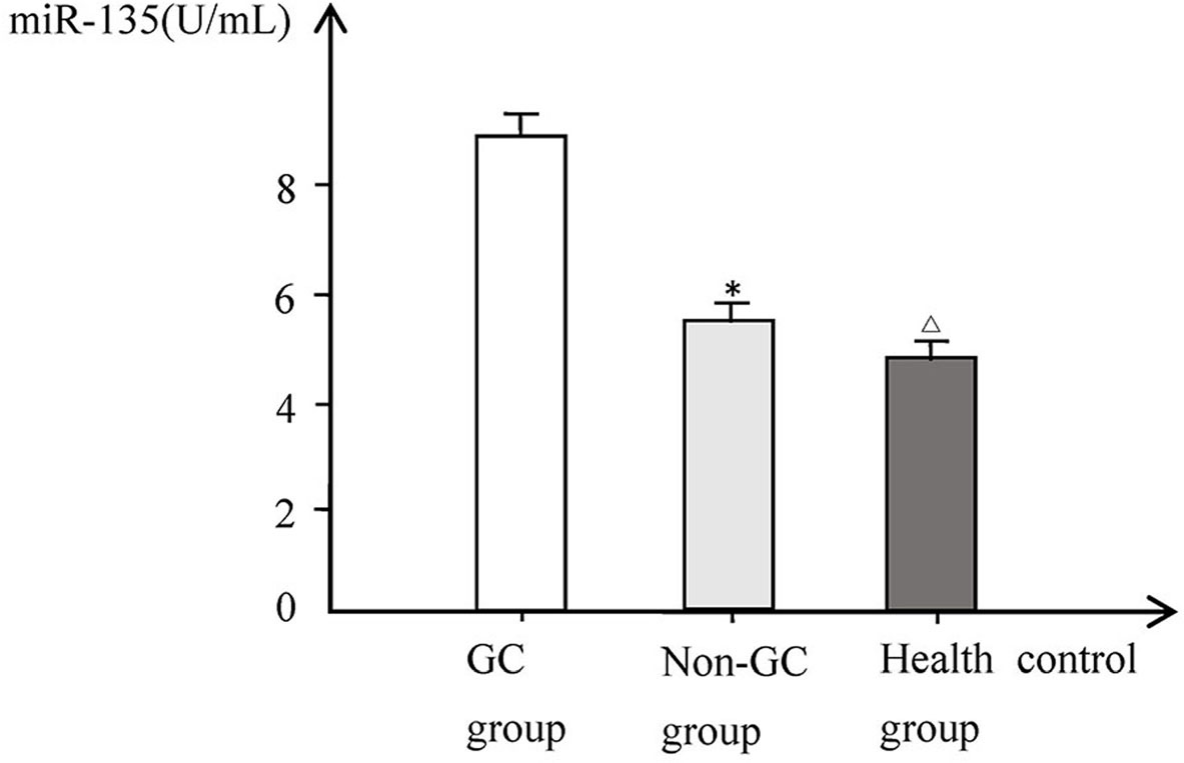
Comparison of serum miR-135 levels in each group

**Fig. 2:**
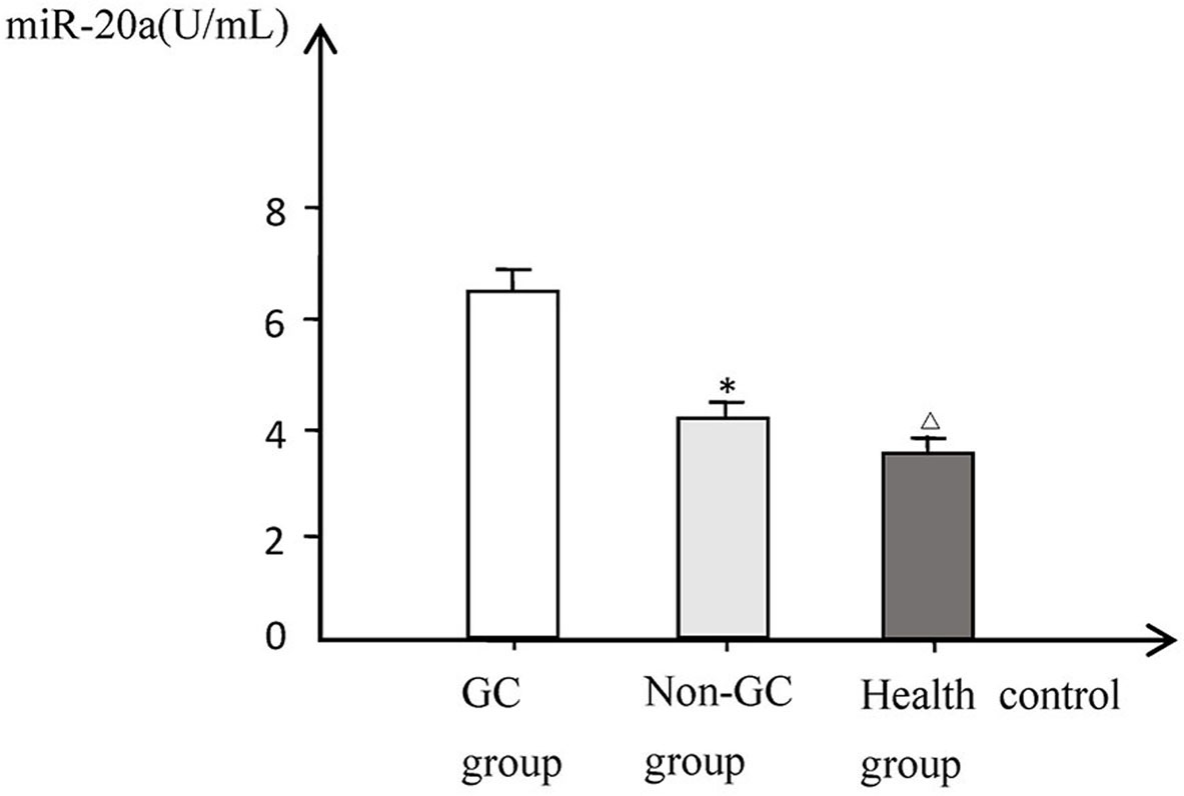
Comparison of serum miR-20a levels in each group

The expression levels of serum miR-135 and miR-20a in patients of the GC group were significantly higher than those of the non-GC group and healthy control group (*P* < 0.01). While no significant difference was found between non-GC group and healthy control group. The expression levels of miR-135 and miR-20a were not significantly correlated with sex and age (*P* > 0.05), but significantly correlated with the degree of GC progression, TNM stage, degrees of differentiation, status of lymph node metastasis, and distant metastasis (Figs. 1 and 2, Table 2).

**Table 2:** Relationship between serum miR-135 and miR-20a expression and clinical characteristics in patients with gastric cancer [(‾*χ* ± *s*), U/mL]

Clinical characteristic	Cases (*n*)	miR-135 expression	*P*	miR-20a expression	*P*
Gender			> 0.05		> 0.05
M	97	7.56 ± 0.57		6.56 ± 0.85	
F	49	7.43 ± 0.54		6.48 ± 0.83	
Age (years)			> 0.05		> 0.05
< 60	88	7.98 ± 0.67		6.64 ± 0.76	
≥ 60	58	8.16 ± 0.69		6.72 ± 0.81	
Degree of gastric cancer progression			< 0.01		< 0.01
Early stage	54	7.96 ± 0.32		5.08 ± 0.46	
Progressive phase	92	9.11 ± 0.34		7.23 ± 0.41	
TNM stage			< 0.01		< 0.01
I-II	76	7.24 ± 0.26		4.93 ± 0.37	
III-IV	70	9.36 ± 0.28		7.51 ± 0.42	
Degrees of differentiation			< 0.01		< 0.01
Well differentiated	94	7.78 ± 0.34		5.56 ± 0.42	
Poorly differentiated	52	9.36 ± 0.41		7.38 ± 0.47	
Lymph node metastasis			< 0.01		< 0.01
No	65	7.43 ± 0.27		5.13 ± 0.49	
Yes	81	9.27 ± 0.32		7.44 ± 0.46	
Distant metastasis			< 0.01		< 0.01
Yes	53	7.51 ± 0.45		5.34 ± 0.66	
No	93	9.43 ± 0.53		7.63 ± 0.72	

### Correlation analysis of serum miR-135 and miR-20a in patients with GC

**Fig. 3:**
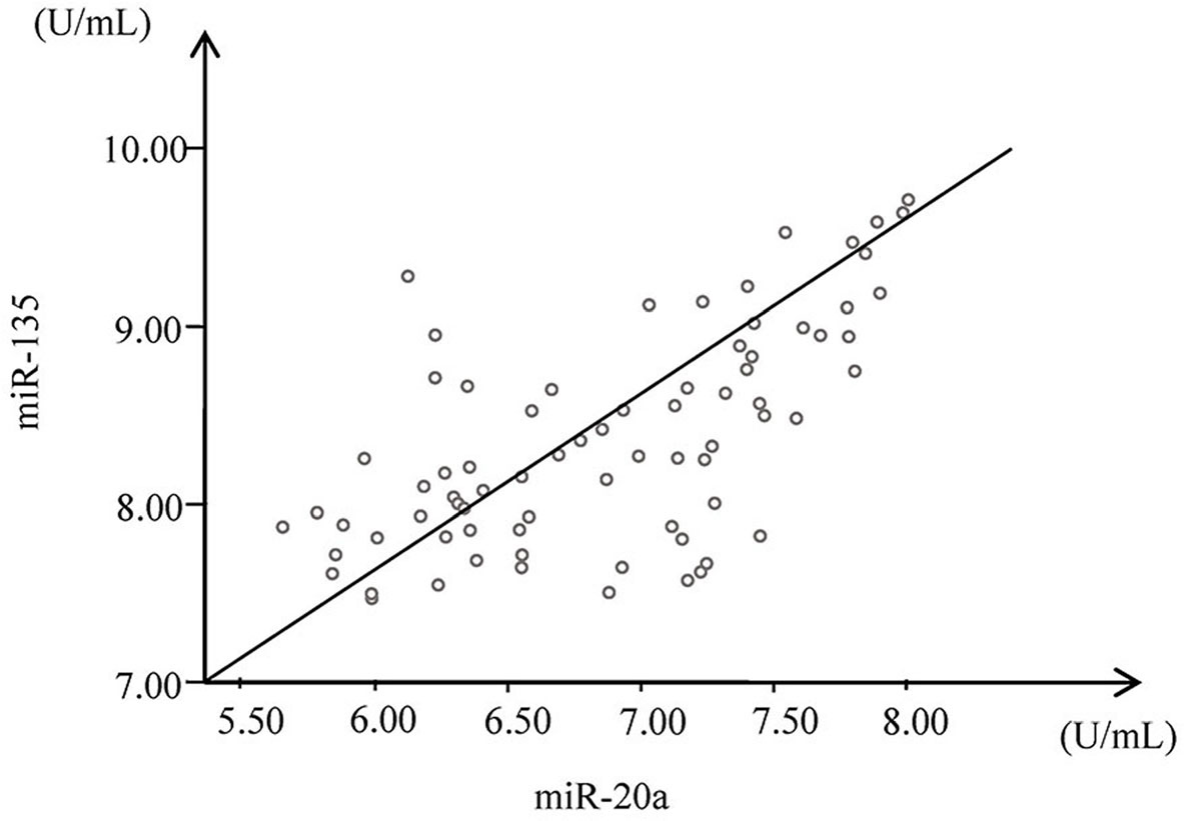
Correlation analysis of serum miR-135 and miR-20a in patients with gastric cancer

As shown in Fig. 3, Pearson’s correlation analysis results showed positive correlations between miR-135 and miR-20a (*r* = 0.634, *P* = 0.000).

### The imaging results of GC examined by MDCT

**Fig. 4:**
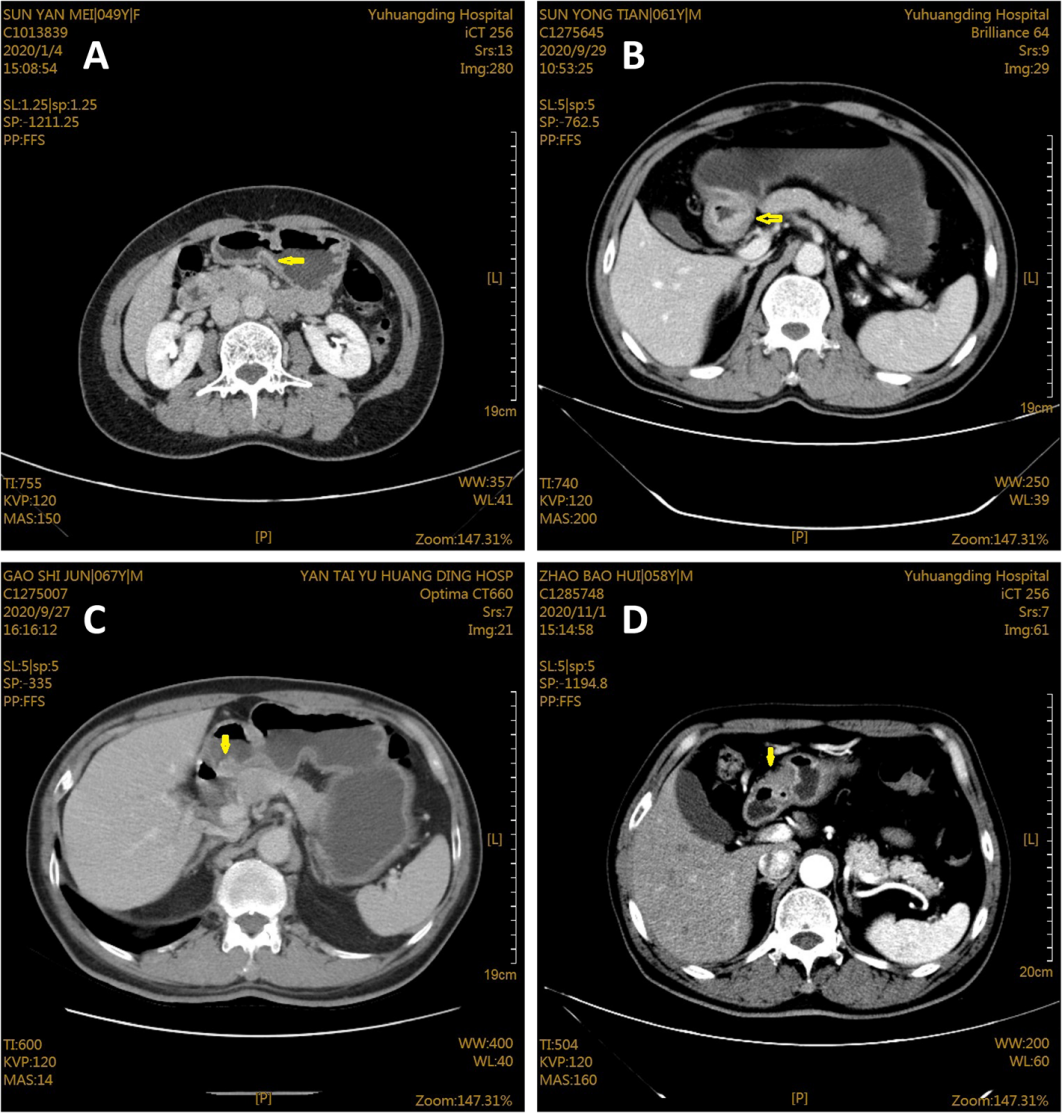
MDCT image

As shown in Fig. 4, patients at T1 stage showed thickening of gastric wall with enhancement of inner layer, as well as visible complete low density band in submucosa (Fig. 4A). While for T2 stage (Fig. 4B), there were thickening of gastric wall, smooth outer edge of gastric wall, and focus breakthrough of low-density zone. For patients with T3 stage, imaging displayed irregular outer serosa margin of thickened gastric wall, blurred space with adipose layer, and presence of nodules (Fig. 4C). Besides, for stage T4 in Fig. 4D, there were blurred serosa and mucosa surface of the gastric wall, unclear adipose layer space, and invasion in the adjacent organs.

### The clinical value of miR-135 and miR-20a combined with MDCT in the diagnosis of GC

**Fig. 5:**
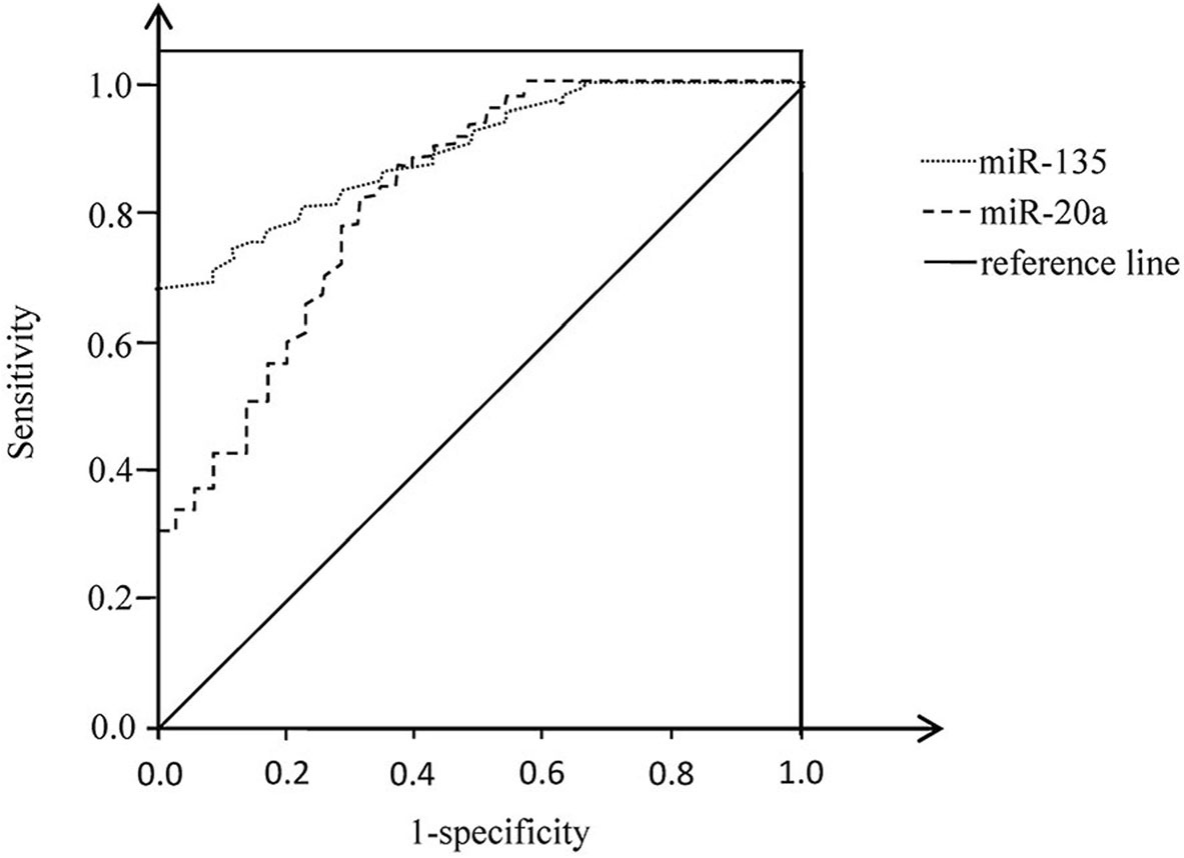
ROC of miR-135 and miR-20a in the diagnosis of gastric cancer

Corresponding results are shown in Fig. 5, Table 3, and Table 4. The ROC analysis results showed that the optimal diagnostic value of miR-135 for GC was 7.56, the AUC was 0.873, and the 95% CI was 0.811-0.935; the optimal diagnostic value of miR-20a for GC was 5.82, the AUC was 0.793, and the 95% CI was 0.697-0.890. These results indicated some disadvantages in single detection, with unsatisfied sensitivity and specificity. The sensitivity and specificity can be improved in combined detection. The sensitivity, accuracy, and negative predictive rate of combined detection were significantly higher than those of single detection (*P* < 0.01).

**Table 3:** Comparison of pathological results of miR-135 and miR-20a in the diagnosis of gastric cancer (*n*)

Items	Pathological results (*n*)		Total
	Positive	Negative	
miR-135			
Positive	114	21	135
Negative	32	82	114
miR-20a			
Positive	116	29	145
Negative	30	74	104
MDCT			
Positive	102	11	113
Negative	44	92	136
Total	146	103	249

**Table 4:** Diagnostic value of miR-135 and miR-20a for gastric cancer

Detection indicator	Sensitivity	Specificity	Accuracy	Positive predictive rate	Negative predictive rate
miR-135	78.08% (114/146)	79.61% (82/103)	78.71% (196/249)	84.44% (114/135)	71.93% (82/114)
miR-20a	79.45% (116/146)	71.84% (74/103)	76.31% (190/249)	80.00% (116/145)	71.15% (74/104)
MDCT	69.86% (102/146)	89.32% (92/103)	77.91% (194/249)	90.27% (102/113)	67.65% (92/136)
Combined detection	90.41% (132/146)	93.20% (96/103)	94.57% (228/249)	94.96% (132/139)	87.27% (96/110)
*P*	< 0.01	> 0.05	< 0.01	> 0.05	< 0.01

## Discussion

The clinical symptoms of GC are not obvious in the early stage. Most of the cases are at middle and late stages when diagnosed, which affects the therapeutic effect and survival of these patients. Early diagnosis and treatment can significantly improve the prognosis of and improve the survival quality of these patients [1]. At present, the commonly used methods for the diagnosis of GC include biopsy and tumor markers. However, these methods have disadvantages [2]. Therefore, it is urgent to find new diagnostic methods for early screening of GC. miRNA is a micromolecule actively secreted by tumor cells, and its expression level changes significantly with the development and regression of tumor. Hence, it plays an important role in the early diagnosis, treatment, and prognosis of most malignant tumors [23, 24]. As evidenced by multiple studies [24–26], the levels of serum miR-135 and miR-20a in GC patients were significantly higher than those in normal tissues, which could promote the proliferation of tumor cells. The results of this study are consistent with their results, which also suggested a poor prognosis of these patients, and the prognosis could be improved after applying targeted therapy. Other studies also showed that the decrease of miR-135 levels could significantly reduce the incidence of postoperative complications in patients with esophageal cancer [27]. Furthermore, MDCT is a rapid, simple, and accurate examination technique. It can show the conditions of gastral cavity, the depth of invasion, and the invasion of adjacent organs and lymph node metastasis. It can be used for accurate determination of the preoperative clinical stage of GC, which has been widely used in the clinical setting [28, 29]. However, MDCT is unable to accurately distinguish the structure of each layer of the gastric wall. While the blurred adipose space caused by perigastric inflammatory reaction may be misdiagnosed as carcinomatous peripheral invasion. Alternatively, there is a lack of abundant adipose layer in patient with relatively lower body weight, which may result in a blurred display of the perigastric adipose space. The pathological tissue of tumor at the early stage may have changed and infiltrated the surrounding tissue, but there may be none significant change in the blood supply. All these reasons will affect the diagnosis results [30, 31].

The results of this study showed that the expression levels of serum miR-135 and miR-20a in GC patients were significantly higher than those of the non-GC patients and health subjects (*P* < 0.01). While no significant difference was found between non-GC patients and healthy controls (*P* > 0.05). Furthermore, the expression levels of these indexes were significantly correlated with the progression of GC, TNM stage, degrees of differentiation, status of lymph node metastasis, and distant metastasis (*P* < 0.01). The results of this study are consistent with the above studies, suggesting that the levels of miR-135 and miR-20a, and IL-1β will increase gradually with the occurrence and development of GC, which may play a significant role in the accurate detection of GC and the severity of GC. Subsequent ROC analysis showed that the AUC of miR-135 and miR-20a for the diagnosis of GC was 0.873 and 0.793 respectively, and the 95% CI was 0.811-0.935 and 0.697-0.890 respectively. The sensitivity, accuracy, and negative predictive rate of the second indexes combined with MDCT in the diagnosis of GC were 90.41% and 94.57% respectively, which were significantly higher than those of each single detection (*P* < 0.01). Correlation analysis showed that there was a positive correlation among serum miR-135 and miR-20a in patients with GC. It suggests that the early diagnosis of GC can be carried out according to the changes of serum miR-135 and miR-20a. Serum miR-135 and miR-20a are expected to be used as new biomarkers for safe and efficient diagnosis of GC, providing some guidance for clinical treatment. A combined detection with MDCT can improve the sensitivity and specificity in the diagnosis of GC, which, to some extent, makes up the deficiency of single detection.

In conclusion, our study suggests abnormal expressions of miR-135 and miR-20a in the serum of GC patients. Imaging with MDCT may help to identify the specific site of the lesion and determine the severity of the disease, which, however, still shows unideal effect when applied alone in the detection of GC. Significantly, combined detection can improve the diagnostic sensitivity of GC at the early stage, contribute to increasing the diagnostic accuracy, and enhance the confidence of physicians and patients. It also has a positive impact on the treatment and prognosis of related patients. Collectively, combined detection of miR-135 and miR-20a with MDCT is of greater significance in the diagnosis of GC clinically. However, findings in our study shall be taken into consideration cautiously and verified in the future due to the limited sample size.

## Data Availability

The datasets used and/or analyzed during the current study are available from the corresponding author on reasonable request.
